# Differences in maternal characteristics and pregnancy outcomes between syphilitic women with and without partner coinfection

**DOI:** 10.1186/s12884-019-2569-z

**Published:** 2019-11-27

**Authors:** Xiao-hui Zhang, Yan-min Chen, Yu Sun, Li-qian Qiu, Dan-qing Chen

**Affiliations:** 1 Department of Women’s Health, Women’s Hospital, Zhejiang University School of Medicine, Xueshi Road 1, Hangzhou Zhejiang, 310006 People’s Republic of China; 2 Department of Obstetrics, Women’s Hospital, Zhejiang University School of Medicine, Xueshi Road 1, Hangzhou Zhejiang, 310006 People’s Republic of China

**Keywords:** Adverse pregnancy outcomes, Syphilis, Couple infection, Treatment

## Abstract

**Background:**

Partner infection is a significant factor in preventing mother-to-child syphilis transmission. We compared pregnancy outcomes between syphilis discordant and syphilis concordant couples.

**Methods:**

We conducted a retrospective study among 3076 syphilis-positive women who received syphilis screening together with their partners during pregnancy. Multivariate analysis was used to explore risks for abnormal outcomes in objects correcting for the major covariate factors. Adjusted odds ratios (OR) and 95% confidence intervals (CI) were estimated to compare pregnancy outcomes between syphilis concordant and syphilis discordant couples.

**Results:**

Overall, 657 of the 3076 women were diagnosed with gestational syphilis and had a syphilis-positive partner, giving a partner concordance prevalence of 21.36%. Women in concordant couples were more likely to have higher parity, more children, late antenatal care and syphilis screening, a lower proportion of latent syphilis, and elevated serologic titers than women in discordant couples (*P* < 0.01 for all). Totally, 10.08% of women had adverse pregnancy outcomes. Multivariate analysis showed partners’ syphilis infection (OR_*adj*_ = 1.44, 95% CI: 1.10–1.89), untreated pregnancy syphilis (OR_*adj*_ = 1.67, 95% CI: 1.15–2.43), and higher maternal serum titers (> 1:8) (OR_*adj*_ = 1.53, 95% CI: 1.17–2.00) increased the risks of adverse pregnancy outcomes. Concordance was associated with increased risk for stillbirth (OR_adj_ = 2.86, 95%CI:1.36–6.00), preterm birth (PTB) (OR_adj_ = 1.38,95%CI:1.02–1.87) and low birth weight (LBW) (OR_adj_ = 1.55, 95%CI:1.13–2.11) compared with discordance. Even among treated women, concordance was associated with increased risk for stillbirth (OR_adj_ = 3.26, 95%CI:1.45–7.31) and LBW (OR_adj_ = 1.52, 95%CI:1.08–2.14). Among women with one treatment course, the risks for PTB(OR_adj_ = 1.81, 95%CI:1.14–2.88) and LBW(OR_adj_ = 2.08, 95%CI:1.28–3.38)were also higher among concordant couples than discordant couples. Nevertheless, there were no significant differences between concordant and discordant couples in risks of stillbirth (OR_adj_ = 2.64, 95% CI: 0.98–7.05),PTB (OR_adj_ = 1.15, 95% CI: 0.76–1.74), and LBW(OR_adj_ = 1.21, 95% CI: 0.78–2.02) among women with two treatment courses.

**Conclusion:**

Male partner coinfection increased the risks for stillbirth, PTB and LBW, particularly when gestational syphilis treatment was suboptimal. However, this risk could be reduced by adequate treatment.

## Background

Syphilis is of particular concern for untreated or inadequately treated pregnant women because of the high prevalence of miscarriage, stillbirth, low birth weight (LBW), preterm birth (PTB) and congenital syphilis [1–4]. In 2016, maternal syphilis was estimated at around 1 million cases globally, and 350,000 women suffered from syphilis-associated unfavorable events [2]. Recently, there has been an upward trend in female syphilis in most areas in the world, including increased maternal syphilis [5–7]. As syphilis is a sexually transmitted infection, factors that can increase re-infection and adverse pregnancy outcomes among women include having more than one sexual partner, a sexual partner’s concurrent infection, and lack of partner treatment during pregnancy [5, 8–10].

Worldwide, tracking partners’ syphilis infection status is urgently needed [11–14]. Among 2 million women of reproductive-age in rural areas in China, the syphilis positive rate for male partners was reported as 14.86% [14]. However, syphilis infection status is unknown for nearly 70% of the partners of pregnant women with syphilis at the national level in China [15]. In 2007, the World Health Organization launched a strategy for the global elimination of congenital syphilis, which stressed active screening and treatment for pregnant women and their partners [16, 17]. Previously, multiple studies based on subjects from underdeveloped regions or high prevalence settings focused on notification and treatment of partners [18–20]. However, data on partners’ infection and the associations with pregnancy outcomes are limited. In this study, we aimed to better understand the syphilis infection status among partners of syphilis-infected pregnant women. We explored differences in maternal characteristics and pregnancy outcomes between pregnant woman with syphilis with and without an infected partner.

## Methods

### Prevention of mother-to-child transmission (PMTCT)

China has conducted a PMTCT project since 2010. In our province, all pregnant women are required to receive syphilis screening via both nontreponemal and treponemal testing during their first antenatal care (ANC)visit and at delivery. Women with positive results or high-risk behaviors are followed-up monthly via serum surveillance. Non-treponemal tests include the tolulized red unheated serum test and the rapid plasma reagin test. Treponemal tests include a *T. pallidum* particle agglutination assay and an enzyme-linked immunosorbent assay. Maternal syphilis is confirmed when a woman has positive results for both nontreponemal and treponemal tests during pregnancy or at delivery. Syphilis-positive pregnant women are immediately offered at least two courses of penicillin treatment. Women who are allergic to penicillin are offered ceftriaxone as a replacement. Safe delivery for syphilitic women is provided by eligible hospitals. Syphilis-positive pregnant women and their infants are routinely followed up by medical staff in local women’s and children’s hospitals. Partner notification, screening, and effective treatment are also recommended.

### Study population and data collection

Data were drawn from the PMTCT surveillance system in Zhejiang province, which is a web-based, mandatory case reporting system for health facilities. Pregnant women who were diagnosed with gestational syphilis from January 2014 to December 2016 were recruited regardless of their pregnancy outcomes. Pregnant women who were syphilis-positive underwent face-to-face interviews with trained medical staff at their local maternal and child health hospital. Verbal consent was obtained from participants. A standard questionnaire was used for data collection, which covered maternal age, education, occupation, marriage, gravidity, parity, syphilis screening and treatment, birth information, and partner’s screening. Additional information was collected from clinical records. Quality control for clinic care and PMTCT information was routinely performed by local and provincial maternal and child health hospitals. All data were collected following approval from the Ethics Committee of Women’s Hospital School of Medicine Zhejiang University. This study conformed to all relevant national laws and provincial regulations.

In this study, stillbirth, PTB, and LBW were analyzed as adverse pregnancy outcomes. Stillbirth was defined as fetal death (≥28 gestational weeks) or death at delivery.PTB was defined as birth before 37 gestational weeks. Birth weight below 2500 g was considered LBW. Women who had stillbirth, PTB, or LBW were included in the incidence of adverse pregnancy outcomes.

### Statistical analysis

Categorical variables were presented as numbers and percentages and compared using chi-square tests. Seropositive pregnant women were divided into two groups based on their partner’s syphilis infection: discordant and concordant couples. A multivariate logistic regression model was used to analyze factors associated with adverse pregnancy outcomes. In further stratified analysis by gestational syphilis treatment, odds ratios (OR) and 95% confidence intervals (CI) were used to compare the risk for adverse pregnancy outcomes between discordant and concordant couples, adjusted by maternal syphilis status, serum titers and first ANC gestational weeks. *P*-values< 0.05 were considered statistically significant. All statistical analyses were performed using SPSS version 13.0(SPSS Inc., Chicago, IL, USA).

## Results

### Maternal characteristics

During the study period, 6502 pregnant women were diagnosed with syphilis infection and 3076(47.31%) partners of those women underwent serological testing. In total, 657 partners had a syphilis-positive result, giving a concordance prevalence of 21.36%. Women in concordant couples were more likely to have higher parity, more children, delayed first ANC visit, higher serum titers, and a lower proportion of latent syphilis than women in discordant couples (Table 1).

**Table 1:** Comparison of maternal characteristics, antenatal care, and syphilis history between pregnant women in syphilis discordant and concordant couples (n, %)

Characteristics	Syphilis discordant (n1 = 2419)	Syphilis concordant(n2 = 657)	χ^2^	*P*
Maternal age				
< 25	590 (24.4)	180 (27.4)	3.81	0.28
25–29	918 (37.9)	239 (36.4)		
30–34	552 (22.8)	154 (23.4)		
≥ 35	359 (14.8)	84 (12.8)		
Ethnic group				
Han	2261 (93.5)	604 (91.9)	1.91	0.17
Minority	158 (6.5)	53 (8.1)		
Education				
College	252 (10.4)	70 (10.7)	0.36	0.84
Middle school	1836 (75.9)	493 (75.1)		
Primary	318 (13.1)	92 (14.0)		
Unknown	13 (0.5)	2 (0.3)		
Occupation				
Farmer	689 (28.5)	187 (28.5)	6.15	0.05
Fix employed	555 (22.9)	178 (27.1)		
Unemployed	1089 (45.0)	266 (40.5)		
Unknown	86 (3.6)	26 (4.0)		
Marriage				
Married	2272 (93.9)	622 (94.7)	0.52	0.47
Others	147 (6.1)	35 (5.3)		
Gravidity				
1	465 (19.2)	115 (17.5)	3.74	0.15
2	735 (30.4)	183 (27.9)		
≥ 3	1219 (50.4)	359 (54.6)		
Parity				
0	1301 (53.8)	287 (43.7)	**33.01**	**< 0.001**
1	921 (38.1)	276 (42.0)		
≥ 2	197 (8.1)	94 (14.3)		
Number of children				
0	1364 (56.4)	315 (47.9)	**21.61**	**< 0.001**
1	907 (37.5)	275 (41.9)		
≥ 2	148 (6.1)	67 (10.2)		
First ANC gestational week				
≤ 12	1350 (55.8)	331 (50.4)	**19.54**	**< 0.001**
13–27	854 (35.3)	230 (35.0)		
≥ 28	215 (8.9)	96 (14.6)		
Previous syphilis infection				
No	1219 (50.4)	320 (48.7)	0.59	0.44
Yes	1200 (49.6)	337 (51.3)		
Diagnose of syphilis infection				
During pregnancy	2265 (93.6)	589 (89.6)	**13.38**	**< 0.001**
At delivery	139 (5.8)	64 (9.7)		
Before pregnancy	15 (0.6)	4 (0.6)		
Syphilis stage				
latent	2090 (86.4)	519 (79.0)	**21.19**	**< 0.001**
I	83 (3.4)	40 (6.1)		
II	12 (0.5)	7 (1.1)		
III	4 (0.2)	5 (0.8)		
Unknown	230 (9.5)	86 (13.1)		
TRUST/RPR titers				
≤ 1:8	2327 (96.2)	614 (93.5)	**9.26**	**0.002**
> 1:8	92 (3.8)	43 (6.5)		

Note: Unknown data were excluded. *TRUST* tolulized red unheated serum test, *RPR* rapid plasma regain

Bold entries have significant values

### Treatment of maternal syphilis

Women with syphilis-negative partners had significantly higher treatment coverage than women with infected partners. Among treated women, no remarkable differences in the proportion of treatment course, length of time between treatment and delivery, time of first treatment, or drugs administered were observed between the two groups. (Table 2).

**Table 2:** Syphilis treatment by partner concordance (n, %)

Variable	Syphilis discordant (n1 = 2419)	Syphilis concordant(n2 = 657)	χ^2^	*P*
Treatment				
Treated	2237 (92.5)	589 (89.6)	**5.85**	**0.02**
Untreated	177 (7.3)	67 (10.2)		
Unknown	5 (0.2)	1 (0.2)		
Treated women: number of treatment courses				
> 2	1799 (80.4)	472 (80.1)	0.02	0.88
1	438 (19.6)	117 (19.9)		
Treated women: duration from treatment to delivery				
≥ 4 weeks	2090 (93.4)	543 (92.2)	2.30	0.13
< 4 weeks	109 (4.9)	38 (6.5)		
Unknown	38 (1.7)	8 (1.4)		
Treated women: Point during pregnancy when received first treatment				
First trimester	1273 (56.9)	330 (1603)	0.37	0.83
Second trimester	347 (15.5)	89 (15.1)		
Third trimester	614 (27.4)	169 (28.7)		
Unknown	3 (0.1)	1 (0.2)		
Treated women: Antibiotics received				
Penicillin	2189 (97.9)	574 (97.5)	< 0.01	0.95
Others	39 (1.7)	10 (1.7)		
Unknown	9 (0.4)	5 (0.8)		

Note: Unknown data were excluded

Bold entries have significant values

### Adverse pregnancy outcomes

Overall, 310 of the 3076 women had adverse pregnancy outcomes, giving an incidence of adverse pregnancy outcomes of 10.08%. In the multivariate analysis (adjusted for maternal age, gravidity, parity, number of children, first ANC gestational weeks, previous adverse pregnancy outcomes, and syphilis stage), factors strongly associated with adverse pregnancy outcomes were partners’ syphilis infection (OR_*adj*_ = 1.44, 95% CI: 1.10–1.89), untreated pregnancy syphilis (OR_*adj*_ = 1.67, 95% CI: 1.15–2.43), and higher serum titers in women (>1:8) (OR_*adj*_ = 1.53, 95% CI: 1.17–2.00).

Further analysis by partners’ infection status showed women in syphilis concordant couples were at higher risk for stillbirth (OR = 3.03, 95% CI: 1.45–6.34) and PTB (OR = 1.43, 95% CI: 1.06–1.94) than women in syphilis discordant couples. Subgroup analysis by pregnancy syphilis treatment showed partner infection increased the stillbirth risk (OR = 3.25, 95% CI: 1.45–7.30), even among women who underwent treatment. Couple coinfection also increased the stillbirth risk among women who received only one treatment course (OR = 3.32, 95% CI: 1.09–10.08). However, among women who received two treatment courses, no increased risks for adverse pregnancy outcomes were observed between women with and without partner infection. Next, when adjusted by maternal syphilis stage, serum titers and first ANC gestational weeks, we observed increased risks in stillbirth (OR_adj_ = 2.86, 95% CI:1.36–6.00), PTB (OR_adj_ = 1.38,95% CI:1.02–1.87) and LBW(OR_adj_ = 1.55, 95%CI:1.13–2.11) among women in syphilis concordant group; increased risks in stillbirth (OR_adj_ = 3.26, 95% CI:1.45–7.31) and LBW (OR_adj_ = 1.52, 95% CI:1.08–2.14) among women in syphilis concordant group and treated; increased risks in PTB(OR_adj_ = 1.81, 95% CI:1.14–2.88) and LBW(OR_adj_ = 2.08, 95% CI:1.28–3.38) among women in syphilis concordant group and with one course treatment. Nevertheless, the risks could be reduced by complete treatment.(Table 3).

**Table 3:** Adverse pregnancy outcomes among treated women: comparison between syphilis discordant and concordant couples (n, %)

Variable	Syphilis discordant	Syphilis concordant	χ^2^	OR (95% CI)	OR_adj_(95% CI)
***Overall***	**n1 = 2419**	**n2 = 657**			
Stillbirth	16 (0.66)	13 (1.98)	9.61	**3.03 (1.45–6.34)**	**2.86 (1.36–6.00)**
Missing	2	1			
PTB	169 (6.99)	64 (9.74)	5.53	**1.43 (1.06–1.94)**	**1.38 (1.02–1.87)**
Missing	4	1			
LBW	101 (4.18)	38 (5.78)	3.41	1.43 (0.98–2.10)	**1.55 (1.13–2.11)**
Missing	16	15			
***Treated***	**n1 = 2237**	**n2 = 589**			
Stillbirth	13 (0.58)	11 (1.87)	8.83	**3.25 (1.45–7.30)**	**3.26 (1.45–7.31)**
Missing	2	0			
PTB	154 (6.89)	50 (8.49)	1.77	1.25 (0.90–1.75)	1.28 (0.92–3.08)
Missing	7	1			
LBW	90 (4.02)	30 (5.09)	1.52	1.31 (0.85–1.99)	**1.52 (1.08–2.14)**
Missing	10	13			
***One course treated***	**n1 = 438**	**n2 = 117**			
Stillbirth	7 (1.59)	6 (5.13)	5.01	**3.32 (1.09–10.08)**	2.61 (0.98–6.92)
Missing	1	0			
PTB	47 (10.73)	19 (16.24)	2.73	1.62 (0.91–2.89)	**1.81 (1.14–2.88)**
Missing	2	1			
LBW	26 (5.94)	10 (8.55)	1.44	1.59 (0.74–3.40)	**2.08 (1.28–3.38)**
Missing	3	8			
***Two course treated***	**n1 = 1799**	**n2 = 472**			
Stillbirth	6 (0.33)	5 (1.06)	2.72	3.20 (0.97–10.52)	2.64 (0.98–7.05)
Missing	1	0			
PTB	107 (5.95)	31 (6.57)	0.24	1.11 (0.73–1.68)	1.15 (0.76–1.74)
Missing	5	0			
LBW	64 (3.56)	20 (4.24)	0.52	1.21 (0.72–2.02)	1.21 (0.78–1.86)
Missing	7	5			

Note: *LBW* low birth weight. *PTB* preterm birth. The odds ratios were adjusted by first ANC gestational weeks, syphilis stage, and serum titers. Unknown data excluded

Bold entries have significant values

## Discussion

The main findings of this study were as follow. Firstly, distribution of maternal characteristics, coverage of pregnancy syphilis treatment, and risks for adverse pregnancy outcomes differed between syphilis discordant and syphilis concordant couples. Women in syphilis concordant couples had higher parity, more children, increased likelihood of being diagnosed and treated late, higher serum titers, and a lower proportion of latent syphilis than women in discordant couples. Secondly, partners’ infection, untreated pregnancy syphilis, and higher serum titers increased the risk for adverse pregnancy outcomes among women with syphilis. Lastly, coinfection of the male partner increased the risks for stillbirth, PTB and LBW, particularly when treatment was suboptimal. However, this risk could be reduced by adequate treatment.

In our study, approximately half of the women’s partners did not receive syphilis screening, although the proportion was greater than corresponding national data for 2013(68.8%); however, it was far below that in 2002–2012data for Shenzhen, China (over 80%) [15, 21]. Despite notification of sexual partners normally being suggested to infected pregnant women in our province, a large gap remains in syphilis testing for partners. However, most women in this study had latent syphilis. This meant that they might have been unaware of the importance of PMTCT or their partner’s status because they had no clinical presentation. In addition, limited knowledge, fears, and stigma have been reported to be crucial barriers to women notifying their partner [13, 18, 22, 23].Consequently, this reduces the coverage of partner testing. In additional, syphilis screening is only freely provided to pregnant women under the PMTCT project, which brings difficulty reaching male partners, partly for economic reasons. Currently, technology-led strategies for partner services, such as email, texts, social or sexual networking sites, and mobile phones have yielded positive results and are recommended globally [24–26].

The concordance prevalence of 21.36% among pregnant women with syphilis in this study was worse than the prevalence reported among Chinese rural women of reproductive age (14.86%) and a study from India(estimated discordant infection rate of84.2%) [14, 27].Globally, few studies have investigated the concordance rate for syphilis infection in couples during pregnancy. Commonly, the concordance rate in men who have sex with men (MSM) is far higher than that among heterosexual couples; for example, 30.2% as reported by the Melbourne Sexual Health Centre [28]. In China, the prevalence of syphilis among MSM married to women was 17.9% [29]. Normally, partners found to be HIV- or HBV-positive ranged from 37.7 to 6.8% for infected pregnant women [30, 31]. However, the above prevalence varies by geographic regions and disease epidemiology. A relative high positive rate in partners in this study should carefully considered the setting of maternal syphilis in Zhejiang, where the large number of migrants has increased the risks for active sexual behavior and sexually transmitted infections [32, 33].

Commonly, adverse pregnancy outcome rates vary from 7% to around 40% [9, 34–36].The total adverse pregnancy outcomes rate in our study was at in the mid-range. Our findings showed partners’ syphilis infection, untreated pregnancy syphilis, and higher maternal serum titers (> 1:8) were risk factors for adverse pregnancy outcomes, which were consistent with previous reports [3, 9, 34]. Importantly, we noticed women in syphilis concordant couples has a nearly three-fold risk for stillbirth and 1.3 to 1.5 -fold risks for PTB and LBW. Even among treated women, the risks remained high when only one treatment course was received. However, when women received adequate treatment, there were no significant differences in adverse pregnancy outcomes between the two groups. Moreover, when odds ratios adjusted by first ANC gestational weeks, syphilis status, and serum titers, we also had the similar findings. A study conducted in Shenzhen, China, indicated that partner infection had a large effect on pregnancy outcomes, with an estimated OR of 2.02 [9]. The increased risk for adverse events in women with syphilis concordant partners may be partly attributable to coinfection in couples. In addition, late ANC, high nontreponemal titers, and inadequate treatment may also lead to adverse outcomes [9, 30, 37, 38].

There were several limitations in this study. First, we had no information on partner characteristics and treatment, despite untreated partners increasing adverse pregnancy outcomes [10, 21]. Second, we only focused on selected adverse pregnancy outcomes (stillbirth, PTB, and LBW) based on available data. Although congenital syphilis was commonly used in previous research, the sample size in our study was too small for robust statistical analyses. Finally, we did not estimate the influence of the high proportion of male partners who did not receive syphilis tests. Given that the proportion was relative to some other studies involved the percentage of partner’s notification and treatment, we did not provide a stratified analysis [15, 17]. The above limitations should be fully considered in further studies.

## Conclusions

The results of this study highlight the need for adequate treatment in pregnancies with syphilis infection to improve the outcomes, particularly when the serologic status of a woman’s partner is unknown or when the partner is known to be affected. Partner screening integrated with early ANC and complete treatment should be prioritized, as has been frequently suggested in previous studies [9, 30, 35–38].

## Data Availability

The dataset generated and analyzed during this study are not publicly available due to confidentiality issues but are available from the corresponding author on reasonable request. Additional, the Mandarin version of the questionnaire used in this study is available from the corresponding author upon reasonable request.

## Abbreviations

ANC: Antenatal care
CI: Confidence intervals
HIV: Human immune deficiency virus
LBW: Low birth weight
MSM: Men who have sex with men
OR: oddsratio
PMTCT: Prevention of mother-to-child transmission
PTB: Preterm birth
SPSS: Statistical package for social science
